# Lifestyle, Age, and Heart Disease Evidence from European Datasets

**DOI:** 10.3390/healthcare13101123

**Published:** 2025-05-12

**Authors:** Samuel Engst, Kristoffer Fangrat, Håkan Lane, Mauro Lombardo

**Affiliations:** 1 Program in Data Engineering, Stockholm Technological Institute, 117 63 Stockholm, Sweden; skengst@gmail.com (S.E.); kristofer.fangrat@icloud.com (K.F.); 2 Department of Computer Science, Johannes Gutenberg University, 55122 Mainz, Germany; 3 Department for the Promotion of Human Science and Quality of Life, San Raffaele Open University, 00166 Rome, Italy; mauro.lombardo@uniroma5.it

**Keywords:** age, body mass index, cardiovascular health, exercise, gender

## Abstract

**Background:** This study examined the interplay between age, exercise, BMI, and cardiovascular health, addressing the growing global health concern of obesity and its link to heart disease. **Methods:** This research utilized data from an Italian dietary survey and the Dutch Longitudinal Internet Studies for the Social Sciences (LISS) panel. Statistical analyses included an ANOVA, linear regression, Mann–Whitney tests, regression with interaction terms, and stratified logistic regression to analyze the impact of age and exercise on BMI and cardiovascular risk. **Results:** This study revealed that BMI increased with age while exercise participation declined, particularly among women. Exercise consistently lowered BMI across all age groups, with no significant interaction between age and exercise. Additionally, age-related increases in cardiovascular risk factors were confirmed, with a higher susceptibility to heart disease in older age. **Conclusions:** These findings highlight the importance of maintaining physical activity to mitigate the risks of obesity and cardiovascular disease. The observed age-specific trends support the development of tailored prevention programs to promote healthier lifestyles across all age groups.

## 1. Introduction

As of 2025, heart disease is the most prevalent cause of death on the planet, with indications that in excess of 30% of annual deaths are connected to various forms of cardiovascular maladies. Body weight is a critical factor, with deviations from a healthy weight being linked to a wide range of cardiovascular diseases (CVDs), type 2 diabetes, and certain types of cancer. The prevalence of obesity and overweight conditions has become a global health crisis, impacting millions of people and burdening healthcare systems worldwide. A multitude of factors influence body weight and body mass index (BMI), including physical activity levels, dietary habits, genetics, and age.

Age is a particularly important factor when discussing overweight. The metabolic rate tends to decrease with age, leading to a natural decline in calorie expenditure over 1grow older, even if their dietary and activity patterns remain consistent. Additionally, age-related changes in muscle mass, hormonal fluctuations, and activity levels further contribute to shifts in body composition. These age-related effects highlight the complexity of weight management and the necessity of examining all contributing factors. On a similar note, it has been noted that CVD is unevenly distributed across age groups [1].

Among the commonly proposed solutions to address issues of unhealthy weight and the accompanying increased risks of heart disease, exercise is frequently emphasized as a key strategy for weight management and improving BMI [2]. Regular physical activity is recommended by healthcare professionals and public health organizations to reduce weight, maintain muscle mass, and improve metabolic health [3]. However, the effectiveness of exercise in directly reducing body weight and BMI is influenced by various factors, including age [4]. For instance, older adults may not experience the same metabolic benefits from exercise as younger individuals, and their capacity to engage in vigorous physical activity may be limited [5].

This study aims to investigate (a) whether the effect of physical activity on BMI depends on age, (b) patterns with regards to age and exercise for both genders, and (c) whether the connection between obesity and heart disease is the same for all ages. By examining these relationships, this research seeks to provide a nuanced understanding of how physical activity and age interact to affect weight management and cardiovascular age groups. Such insights could enhance strategies for mitigating the health risks associated with obesity, particularly among different age groups.

There are three research questions:

RQ1: What is the effect of exercise on obesity and BMI in different age groups?

RQ2: How much do people of various ages exercise, as well as with respect to their gender?

RQ3: What is the connection between BMI and cardiovascular illnesses at different ages?

## 2. Theory

### 2.1. Exercise and BMI by Age

Body mass index (BMI) is a widely used measure to assess body weight relative to height, and it is often used to categorize individuals into different weight status categories, such as underweight, normal weight, overweight, and obese. For adults, the relationship between exercise and BMI is well documented. Regular physical activity of at least 150 min per week helps maintain a healthy weight, reduce body fat, and improve overall well-being. Additionally, muscle-strengthening activities should be performed at least two days a week [6].

In older adults, maintaining a healthy BMI through 75–150 min of exercise per week is essential for preventing chronic diseases and enhancing functional capacity. Balance and strength training exercises are also recommended to prevent falls and improve functional independence [6]. 

### 2.2. Exercise and Age

Children and adolescents are generally more active than adults, with physical activity being a crucial component of their growth and development. Studies have found that regular physical activity in this age group improves motor skills, cognitive function, and overall health. A systematic review highlighted that children and adolescents benefit from a variety of physical activities, including team sports, dance, and age-appropriate resistance training [6]. Encouraging active play and participation in diverse sports can help establish lifelong fitness habits.

Young adults often experience a shift in lifestyle due to work, education, and social obligations, which can impact their exercise habits. Despite these challenges, maintaining a consistent fitness routine is vital for stress reduction, increased energy, and overall health. Research indicates that young adults prefer high-intensity interval training (HIIT), circuit training, and bodyweight exercises that can be performed anywhere, anytime [7]. These efficient and adaptable exercise programs help young adults achieve their wellness goals without compromising their busy schedules.

For middle-aged adults, regular physical activity is essential for preventing chronic diseases and maintaining functional capacity. This age group often faces time constraints due to work and family responsibilities, making it important to incorporate exercise into their daily routines. Studies have shown that middle-aged adults benefit from a combination of aerobic activities, strength training, and flexibility exercises. Tailoring exercise programs to individual needs and preferences can enhance adherence and promote long-term health [8].

### 2.3. Overweight and Cardiovascular Disease at Different Ages

A high body mass index (BMI) is a well-established risk factor for various cardiovascular diseases, including angina, heart attacks, and strokes. The relationship between a high BMI and these conditions varies across different age groups, influenced by physiological, lifestyle, and genetic factors.

Angina, characterized by chest pain due to reduced blood flow to the heart, is often a precursor to more severe cardiovascular events. Studies have shown that a high BMI is associated with an increased risk of angina. For instance, a study on Norwegian patients with suspected stable angina found that obese men had a significantly higher risk of acute myocardial infarction (AMI) and cardiovascular death than their normal-weight counterparts [9]. This association underscores the importance of managing BMI to prevent the onset of angina and subsequent cardiovascular complications.

Heart attacks, or myocardial infarctions, occur when a blocked artery prevents blood from reaching a part of the heart muscle. A high BMI has been implicated as a significant risk factor for heart attacks. A Mendelian randomization study in European adults demonstrated that a higher BMI is causally associated with an increased risk of heart attacks [10]. The study found that the risk of heart attacks increased by 0.8% per 1-SD (4.5 kg/m^2^) increase in BMI. This highlights the critical need for weight management strategies to reduce the incidence of heart attacks, particularly in populations with a high BMI.

Strokes, which occur due to an interruption of blood flow to the brain, are another major cardiovascular event linked to a high BMI. A meta-analysis examining the relationship between BMI and stroke risk found that a higher BMI was associated with an increased overall risk of stroke [11]. The study reported that overweight and obese individuals had a significantly higher risk of stroke than those with a normal BMI. This association was particularly strong for ischemic strokes, which account for the majority of stroke cases. The findings suggest that maintaining a healthy BMI is crucial for stroke prevention across all age groups.

The impact of a high BMI on the risk of angina, heart attacks, and strokes varies across different age groups:

1. Children and Adolescents: While cardiovascular diseases are rare in this age group, a high BMI can set the stage for future cardiovascular risk. Early intervention and weight management are essential to prevent the development of cardiovascular conditions later in life.
2. Young Adults: In young adults, lifestyle factors such as diet and physical activity play a significant role in BMI and cardiovascular risk. A high BMI in this age group is associated with an increased risk of developing cardiovascular diseases, emphasizing the need for healthy lifestyle choices.
3. Middle-Aged Adults: The risk of cardiovascular diseases increases significantly in middle-aged adults with a high BMI. This age group often experiences the cumulative effects of a long-term high BMI, making weight management and regular health check-ups critical.
4. Older Adults: Older adults with a high BMI are at the highest risk of cardiovascular diseases. Age-related changes in the cardiovascular system, combined with a high BMI, contribute to the increased prevalence of angina, heart attacks, and strokes in this population. Comprehensive management strategies, including medication, lifestyle changes, and regular monitoring, are essential for this age group.

### 2.4. Regression with Interaction

Regression with interaction terms is an advanced statistical technique employed to investigate whether the effect of one predictor variable on a dependent variable changes depending on the level of another predictor variable. This method is particularly beneficial in fields such as psychology, epidemiology, and economics, where it is critical to understand the intricate interdependencies between variables. Interaction terms are created by multiplying two predictor variables, allowing researchers to explore how the relationship between one predictor and the outcome variable varies as a function of another predictor.

The inclusion of interaction terms in regression models provides deeper insights into complex relationships that may be overlooked when considering only main effects. For example, in psychological research, interaction effects can elucidate the conditions under which specific behaviors emerge. In epidemiology, they can reveal how various risk factors synergistically influence health outcomes [12,13].

The estimation of interaction effects typically involves moderated multiple regression (MMR), which includes the interaction term in the regression model. The coefficients of these terms are then interpreted to understand how the relationship between predictors and the outcome variable changes at different levels of the interacting variables. It is advisable to center the predictor variables before creating the interaction term to reduce multicollinearity and enhance interpretability [14].

### 2.5. Stratified Logistic Regression and Odds Ratios

Logistic regression is a widely used statistical method for modeling the relationship between a binary dependent variable and one or more independent variables. It is particularly useful in medical and epidemiological research for assessing the association between risk factors and health outcomes. One important application of logistic regression is the calculation of odds ratios (ORs), which quantify the strength of the association between predictors and the outcome [15]. Stratifying these odds ratios into age groups allows for a more nuanced understanding of how age influences the relationship between predictors and the outcome [16].

To perform logistic regression with odds ratios stratified into age groups, the subjects are divided into age groups (e.g., 0–20, 21–40, 41–60, and 61+ years) to analyze the effect of age on the relationship between predictors and the outcome. Separate logistic regression models for each age group are obtained. The logistic regression model can be expressed as follows:

$$logit(P(Y=1))=\beta_{0}+\beta_{1}X_{1}+\beta_{2}X_{2}+\cdots +\beta_{k}X_{k}$$

where P(Y = 1) is the probability of the outcome occurring; X_1_, X_2_,…, X_k_ are the independent variables; and β_0_, β_1_,…, β_k_ are the coefficients. The odds ratio (OR) for a predictor X_i_ is given by

$${\mathrm{OR}}_{i}=e^{\beta_{i}}$$

where β_i_ is the coefficient of X_i_ in the logistic regression model. The odds ratio measures the increase in the risk percentage for the outcome when the value is increased by one unit and can be stratified for each age group [17].

### 2.6. The LISS Panel

The Longitudinal Internet Studies for the Social Sciences (LISS) panel is a high-quality online research infrastructure in the Netherlands, managed by Centerdata at Tilburg University. Established in 2007 with funding from the Netherlands Organization for Scientific Research (NWO), the LISS panel provides a representative sample of the Dutch population for scientific research. The panel consists of approximately 7500 individuals from 5000 households who complete monthly online surveys on a wide range of topics.

The LISS panel collects data through various surveys and experiments, which are designed to address diverse research questions in the social sciences. The data collection process follows a “privacy-by-design” approach, ensuring that all data are collected and stored in accordance with the FAIR principles (Findable, Accessible, Interoperable, and Reusable). The panel members are recruited through a probability-based sampling method, ensuring that the sample is representative of the Dutch population.

The LISS Data Archive contains three main sources of data:

1. Background Variables: Monthly updated socio-economic and demographic information.
2. LISS Core Study: An annual longitudinal survey consisting of multiple questionnaires covering a broad range of topics, including health, religion, social integration, family, work, personality, politics, and economic situation.
3. Assembled Studies: Surveys and experiments conducted as paid or externally funded assignments.

Researchers and policymakers worldwide can access the LISS data through the LISS Data Archive. The archive allows users to download existing datasets, link LISS data to their own research data, and enrich their datasets with registry microdata from Statistics Netherlands (CBS). The LISS panel is also part of ODISSEI (Open Data Infrastructure for Social Science and Economic Innovations), a sustainable research infrastructure that supports social science research in the Netherlands.

## 3. Materials and Methods

### 3.1. Italian Dataset

This dataset, derived from an ongoing online survey at a Rome medical center, examines dietary patterns and lifestyle factors among patients. Participants completed a 30 min online questionnaire before their initial visit. The study included adults (18+) fluent in Italian, who provided informed consent.

#### 3.1.1. Survey Methodology

Participants completed an anonymous online survey before their visit, accessible via any internet-enabled device.

#### 3.1.2. Data Collection Process

The data collection process involved the following steps:

- Pre visit: Online survey on eating habits, food preferences, sleep, and physical activity.
- Visit: Bioelectrical impedance analysis (BIA) and anthropometric measurements.
- Follow-up: Seven-day food diary based on prescribed diet plans.

#### 3.1.3. Sampling and Study Size

From November 2023, over 3000 surveys were collected, with post hoc power calculations confirming a sufficient sample size. The participants were grouped into age groups of 7 years: 18–24, 25–31, 32–38, 39–45, 46–52, 53–59, 60–66, and 67–73. These age groups were selected to balance statistical power with granularity, enabling the identification of subtle age-related trends in exercise habits and BMI. This stratification ensured an adequate number of observations in each group while capturing meaningful differences between life stages.

#### 3.1.4. Ethical Considerations

This study was approved by the IRCCS San Raffaele Ethics Committee (RP 23/13) and adhered to the Declaration of Helsinki. It was also registered on ClinicalTrials.gov (NCT06654674).

#### 3.1.5. Dietary and Physical Activity Assessment

The survey assessed eating habits, meal patterns, food preferences, 24 h dietary recall, and beverage consumption. Physical activity was assessed through a self-administered online question asking whether participants engage in regular physical activity, defined as at least 150 min of moderate-intensity exercise per week (yes/no format).

### 3.2. LISS

Thirteen waves of the health cohort between 2008 and 2022 were extracted and merged based on id, such that no duplicates remained. The responses were derived based on the respondents’ entered code into the questions listed in Table 1. In the LISS panel, physical activity was self-reported through a yes/no item indicating whether the respondent was physically active, without further details on intensity, frequency, or duration.

The BMI value for each person was derived through the standard formula:

$$BMI=10,000\times \frac{weight/kg}{(height/cm{)}^{2}}$$

Age was divided into the intervals ≤25, 26–50, 51–75, and above 75 years.

### 3.3. Statistics

The influence of age on BMI and exercise was measured through an Analysis of Variance (ANOVA) and linear regression, with a significant coefficient indicating a trend from younger to higher age groups. We used Mann–Whitney tests to determine whether the genders had any significant differences.

For the analysis of the effect of exercise on BMI based on age, regression with interaction terms of the form

BMI ~ exercise + age + exercise age

was used. A significance level of alpha = 0.05 was used as the measure of significance.

The strength of the association between BMI and CVD was estimated through logistic regression models for each combination of condition (angina, heart attack hypertension, high cholesterol, and stroke) and age subgroup. Odds ratios were employed as benchmarks.

## 4. Results

### 4.1. Description of Italian Dataset

The overall mean values/distributions for the studied variables in the Italian dataset are shown in Table 2.

### 4.2. BMI and Exercise by Age Group

We show the mean BMIs of each gender and age group, together with the fraction of respondents answering yes to the question of exercise, in Table 3.

**Figure 1:**
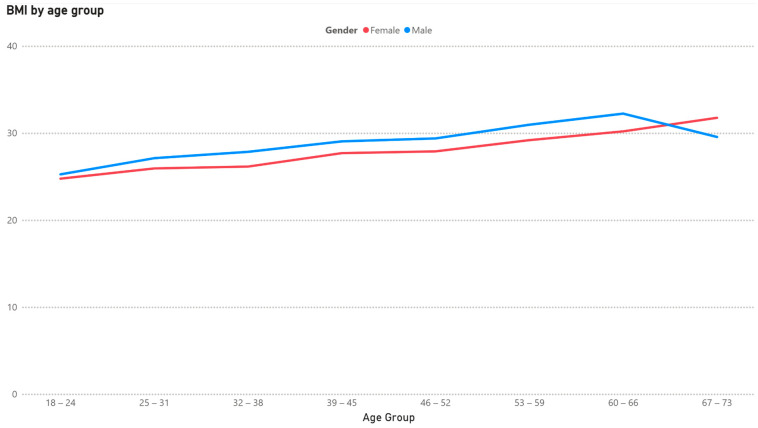
Mean BMI by age group for females and males.

**Figure 2:**
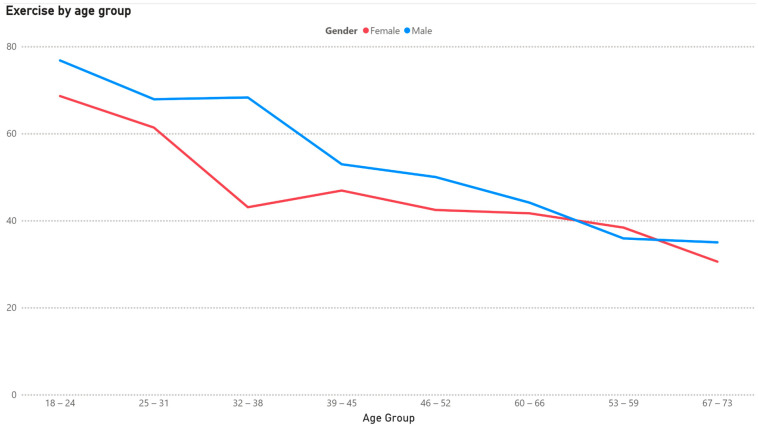
Share of population engaging in exercise by gender and age.

There is a clear shift towards a higher BMI and less exercise from younger to higher ages, as shown in Figure 1 and Figure 2.

### 4.3. Interaction Between Exercise, BMI, and Age

**Figure 3:**
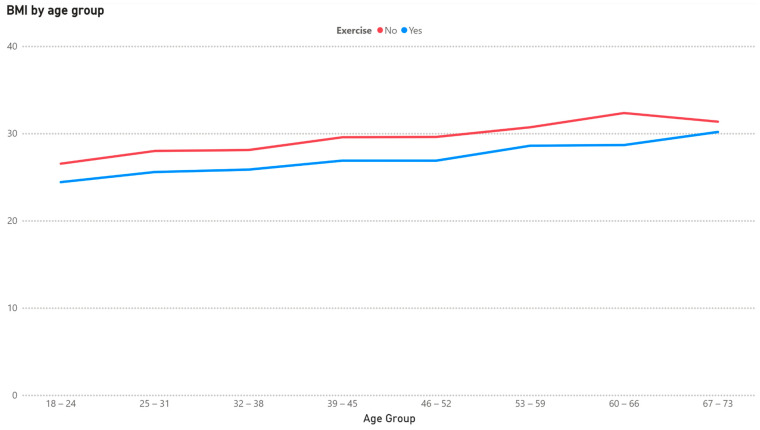
BMI based on exercise by age group.

The mean BMI of each cohort (exercise/non-exercise) stratified by age group is shown in Figure 3.

There is a progression towards a higher BMI as age rises, and, in each group, the mean is lower for the group engaging in exercise. We present the results of the regression with interaction terms in Table 4.

It could be observed that both exercise and age have an influence but that the effect of exercise is not dependent on age.

### 4.4. Descriptives LISS Set

The sample consisted of 11,976 persons recruited between the years 2008 and 2021. We list the mean/distribution for each of the studied features of the Dutch cohort in Table 5.

### 4.5. Heart Disease by Age Group

We show the prevalence of three heart disease conditions based on age, gender, and type of malady in Table 6.

The onset of higher risk appears to begin earlier for males (46–55) than for females (56–65).

### 4.6. Connection Between Heart Disease and BMI by Age Group

**Figure 4:**
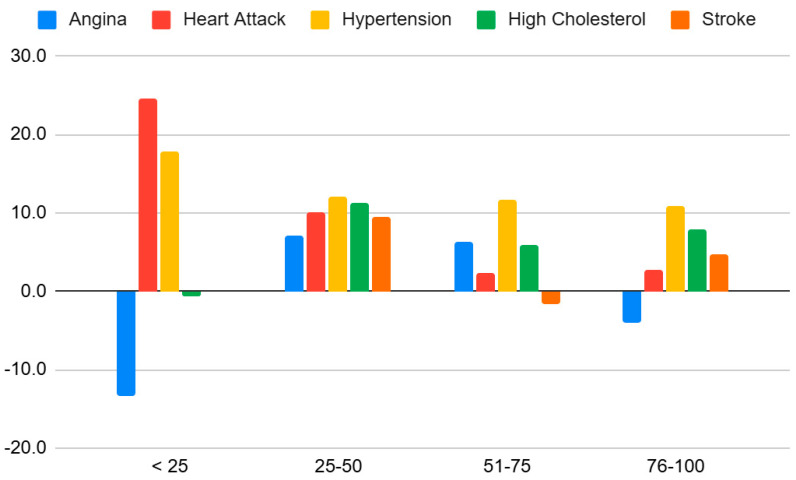
Odds ratios by age group and condition.

The odds ratios for the connection between BMI and heart disease measured for five conditions (angina disease, heart attack, hypertension, high cholesterol, and stroke) are presented in Figure 4 and Table 7.

The effects are clearly most pronounced in the younger and middle ages.

The effects are the most consistent for hypertension at all ages and for all conditions in the middle ages.

## 5. Discussion

### 5.1. Age and Gender Differences

The result clearly shows that BMI tends to increase with age, in line with previous research [18]. With age, metabolism often slows down, making it easier to gain weight [18]. Hormonal changes, especially in women after menopause, can affect weight distribution and increase the risk of weight gain [19].

The data show a sharp decline in exercise with increasing age, especially for women, and this should be a cause of concern in the healthy aging discussion and could be explained by multiple factors. Li et al. [20] found that recovery from muscle damage takes a considerably longer time for older individuals, while a set of focus group interviews revealed that the motivation to be physically active alone is much lower for individuals aged 60 or above [21]. This indicates that arranging group training opportunities targeted at the retired could serve as a way to achieve better weight management and cardiovascular health, something that was recommended by Box et al. [22]. The fall in exercise frequency is especially sharp for women entering their 30s, while men tend to remain active into later stages in life. This gender gap was confirmed through the Eurobarometer study [23].

Additionally, the LISS data indicate that the prevalence of cardiovascular diseases increases with age, a commonly observed trend [24]. The results demonstrate age-related increases in cardiovascular risk factors, notably angina, heart attack, hypertension, and high cholesterol. This indicates a heightened vulnerability to heart disease with advancing age, particularly in the <25 age group, which warrants further investigation.

### 5.2. Exercise vs. Non-Exercise

Exercise was associated with a lower BMI across most age groups, reflecting the expected effects of energy balance and an increased muscle mass [25,26,27]. The regression with interaction terms confirmed that both age and exercise independently influence BMI, but their interaction was not statistically significant in most cases. This indicates that the BMI-lowering effect of physical activity is relatively stable across age groups rather than being more or less pronounced with advancing age. The marginal effect observed in the 60–67 group (*p* = 0.07) may merit further investigation.

A comprehensive meta-analysis of randomized clinical trials indicated that engaging in just 30 min of aerobic exercise per week led to modest reductions in body weight, waist circumference, and body fat, while more weight loss required more than 150 min of moderate-intensity work [28]. The findings confirmed that exercise plays a crucial role in improving body composition, emphasizing its importance in managing weight and overall health in these populations [29]. When considering the optimal form of exercise, it is crucial to align the activity with the desired outcome, be it weight management, muscle development, or enhanced metabolic function.

Research consistently demonstrates the efficacy of various exercise modalities tailored to specific objectives. For calorie expenditure and cardiovascular enhancement, endurance training, encompassing activities like running, cycling, and swimming, proves highly effective. For example, research has demonstrated that exercise and physical activity have effects on weight loss and maintenance [5].

Conversely, strength training, through weightlifting, bodyweight exercises, or resistance bands, is fundamental for increasing muscle mass, which in turn elevates the resting metabolic rate. Research has shown that physical activity has an influence on BMR [20]. High-intensity training (HIIT) emerges as a time-efficient strategy, maximizing calorie burn within brief periods while yielding prolonged metabolic benefits through the “afterburn” effect. HIIT has been shown to maximize calorie burn and provide metabolic benefits. Research shows that exercise, energy balance, and body composition are all linked together [25]. Finally, mixed modalities, such as CrossFit, team sports, and martial arts, offer a comprehensive approach, integrating strength and cardiovascular components to deliver well-rounded fitness improvements. Research demonstrates the benefits of mixed modalities for overall fitness. Also, it is important to understand the effects of obesity [30].

### 5.3. Connection to Heart Disease

The logistic regression outcomes were highly conclusive for hypertension being the main consequence of a high BMI, much in line with previous findings [31]. The odds ratios stratified by age showed that the association between a high BMI and cardiovascular outcomes is stronger in younger and middle-aged adults, particularly for hypertension and heart attack. In contrast, the associations appear weaker or inconsistent in the older age group, possibly due to survival bias or age-related physiological changes. These results indicate that early weight management may be particularly effective in reducing long-term cardiovascular risk. The mechanisms for this connection include adipose tissue dysfunction, vasoconstriction, and sodium [32]. The inverse association between BMI and some of the conditions at younger ages calls for reflection and is most likely caused by the high number of individuals with underweight in these groups. On inspection, 11% of the respondents aged under 25 had a BMI less than 18.5, while the corresponding share was only 1.5% for the other groups combined. This U-shaped correlation, where the lower spectrum of BMI values also carries a risk of adverse cardiovascular outcomes, has been observed in multiple studies.

### 5.4. Study Limitations

The study design has some limitations. Some variables, such as physical activity levels and self-reported eating habits, may lack detailed standardization. Although eating habits were recorded as binary variables (yes/no), other aspects, such as the frequency and intensity of exercise, may be subject to variations in interpretation, resulting in inconsistencies in data collection. In addition, the measurement of physical activity was based on self-reported yes/no responses, with limited detail on the frequency, duration, or intensity of exercise, particularly in the LISS dataset. This may reduce the accuracy of the measurement. In addition to self-report bias, potential selection bias must be considered, especially for the Italian dataset. Participants were recruited from a medical center in Rome, which could lead to a sample with higher health awareness or existing health problems, thus limiting the generalizability of the results to the broader Italian population.

This study used self-reported data for the main health and lifestyle factors. This introduces the risk of recall bias and social desirability bias, where participants may unintentionally misreport food intake, exercise habits, or weight information to align with perceived health norms [33]. The sample size should be considered sufficient; some groups may have too few observations.

BMI (body mass index) is a widely used metric to calculate body weight relative to height. However, it does not distinguish between muscle mass and body fat. As a result, individuals with a high muscle mass, such as athletes, may be classified as overweight or obese despite having a low body fat percentage. Conversely, older adults or individuals with sarcopenia (age-related muscle loss) may fall within a “normal” BMI range while still having an unhealthy body composition with excess fat and reduced muscle mass [34]. This limitation highlights the need for alternative or complementary measures, such as body fat percentage, waist-to-hip ratio, muscle mass assessment, or waist-to-height ratio. Using these alongside other metabolic markers like blood glucose levels or cholesterol levels would assist in providing a more accurate and personal health risk assessment, as two individuals with the same BMI may have different health risks depending on the previously mentioned factors [35].

### 5.5. Future Research Directions

A longitudinal approach, tracking BMI alongside other health indicators over time, would provide a more comprehensive picture of an individual’s health trajectory. Further research is needed to better understand the long-term effects of exercise on BMI, muscle mass, and fat distribution. Longitudinal studies tracking individuals over extended periods would provide valuable insights into how different exercise regimens influence body composition over time [5]. Additionally, personalized exercise programs could be explored to determine how genetic factors, metabolic rate, and lifestyle influence the effectiveness of various types of physical activity. Tailoring exercise routines based on these factors may optimize weight management and overall health outcomes. Another important area of research is the integration of exercise with other lifestyle interventions. Investigating the combined effects of physical activity, diet, sleep quality, and stress management could lead to more effective strategies for maintaining a healthy BMI and body composition. Beyond BMI, future research should also explore alternative or complementary metrics for assessing health risks, such as waist-to-height ratio, waist circumference, and body fat percentage. These metrics would provide accurate assessments for groups where BMI may provide incorrect health assessments, such as athletes, the elderly, or individuals with metabolic-related medical issues. Understanding how these alternative indicators respond to exercise and other interventions could improve accurate identification of at-risk individuals and assist in providing appropriate assistance to prevent potential ailments. Lastly, advancements in technology provide an opportunity to enhance research accuracy. Objective measurement tools, such as wearable fitness trackers and AI-driven health monitoring systems, could be utilized to better quantify activity levels and assess their impact on body composition. These technologies may offer more precise and individualized recommendations for improving health through exercise.

A novel contribution of this study is the combined use of two independent datasets from different European populations—Italy and the Netherlands—allowing for a cross-validation of trends in different demographic and health contexts. Furthermore, the stratified analysis of age, exercise participation, and cardiovascular outcomes provides a nuanced perspective on the interaction of these variables. This multidimensional approach offers new insights into the differential impact of physical activity across age groups, with implications for the design of age-specific health interventions. An important direction for future research is to investigate whether individuals with advanced age and a high BMI can effectively reduce their cardiovascular risk through targeted lifestyle changes. Although our results confirm strong associations, longitudinal studies are needed to determine whether improved dietary habits and sustained physical activity can reverse the cardiovascular burden in older populations. Clarifying this reversibility would have significant implications for public health policies and personalized prevention strategies.

## 6. Conclusions

This study reveals a clear trend of increasing BMI with age, accompanied by a marked decline in exercise participation, especially among women. These findings underline the importance of maintaining regular physical activity and healthy eating habits to reduce the risks of obesity and cardiovascular disease. The observed age-specific trends support the development of tailored prevention programs and public health interventions aimed at promoting healthy lifestyles in all age groups. We also establish that the susceptibility to heart disease associated with being overweight varies with age and that overweight is more consistently linked to hypertension, a known precursor to many adverse cardiovascular outcomes [31]. Encouraging physical activity, particularly in the 60–67 age group, may be particularly effective. These findings provide a solid basis for further research into the mechanisms driving these trends, including the role of underweight as an additional risk factor.

## Figures and Tables

**Table 1:** Questions on health.

Question	Follow-Up	Unit
How tall are you?		cm
How much do you weigh, without clothes or shoes?		kg
Has a physician told you this last year that you suffer from…		Yes/No
	angina, pain in the chest?	
	a heart attack including infarction or coronary thrombosis or another heart problem including heart failure?	
	a stroke or brain infarction or a disease affecting the blood vessels in the brain?	

**Table 2:** Overall measures for Italian dataset.

Feature	Mean/Distribution
Age	39.72 years
BMI	27.63 kg/m
Exercise	53.12%

**Table 3:** Mean BMI and percentage of people engaging in exercise by gender and age group.

Age Group	BMI Mean Males/Females		Percentage of Population Engaging in Exercise Males/Females	
18–24	25.26	24.76	76.80	68.61
25–31	27.12	25.94	67.88	61.36
32–38	27.85	26.15	68.29	43.07
39–45	29.05	27.70	52.94	46.90
46–52	29.39	27.90	50.00	42.44
53–59	30.96	29.19	35.90	38.39
60–66	32.24	30.20	44.12	41.67
67–73	29.56	31.76	35.00	30.56

**Table 4:** Coefficient interaction regression for BMI vs. exercise and age.

Term	Estimate	Pr (>|t|)
exercise	−2.13	0.0023 **
age_group [25, 32)	1.76	0.016 *
age_group [32, 39)	1.47	0.032 *
age_group [39, 46)	2.94	2.5 × 10^−5^ ***
age_group [46, 53)	3.22	3.6 × 10^−6^ ***
age_group [53, 60)	4.33	8.7 × 10^−9^ ***
age_group [60, 67)	6.26	1.2 × 10^−13^ ***
age_group [67, 74)	4.80	1.5 × 10^−6^ ***
exercise × age_group [25, 32)	−0.44	0.61
exercise × age_group [32, 39)	−0.03	0.97
exercise × age_group [39, 46)	−0.55	0.53
exercise × age_group [46, 53)	−0.76	0.39
exercise × age_group [53, 60)	−0.42	0.68
exercise × age_group [60, 67)	−2.05	0.07
exercise × age_group [67, 74)	0.97	0.54

Note: * *p* < 0.05, ** *p* < 0.01, *** *p* < 0.001.

**Table 5:** Descriptive LISS data from the Netherlands.

Feature	Mean/Distribution
Age	44 years
BMI	25.0 kg/m^2^
Angina	1.9%
Heart Attack	1.4%
Hypertension	11.9%
High Cholesterol	7.5%
Stroke	0.7%

**Table 6:** Share of population.

Age Group	Angina% Male/Female		Heart Attack% Male/Female		Stroke% Male/Female	
16–25	0.5	1.5	0.2	0.2	0.0	0.0
26–35	1.4	1.2	0.5	0.2	0.3	0.4
36–45	0.8	1.4	0.6	0.2	0.1	0.1
46–55	2.6	1.4	1.6	0.3	0.4	0.4
56–65	2.9	1.6	3.7	1.7	2.0	1.0
66–75	3.7	3.4	4.8	3.1	2.0	1.9
76–85	6.4	3.3	7.8	6.2	3.0	3.1
86–95	5.8	2.0	10.0	5.3	5.0	N/A ^a^

^a^ Not enough respondents.

**Table 7:** Odds ratios by age group and condition.

	<25	25–50	51–75	76–100
Angina	−13.4	7.2 **	6.4 **	−3.9
Heart Attack	24.6 ***	10.0 *	2.3	2.7
Hypertension	17.8 ***	12.1 ***	11.6 ***	10.8 ***
High Cholesterol	−0.7	11.3 ***	5.8 ***	8.0 *
Stroke	0.0	9.6	−1.7	4.7

Note: * *p* < 0.05, ** *p* < 0.01, *** *p* < 0.001.

## Data Availability

The raw data supporting the conclusions of this article will be made available by the authors on request.
